# The relationship between structural characteristics of long-term care institutions and their initial operating budgets

**DOI:** 10.3389/fpubh.2025.1669451

**Published:** 2025-10-02

**Authors:** Xiaojing Wang, Peter C. Coyte

**Affiliations:** ^1^Department of Business Administration, School of Management, Shanghai University of Engineering Science, Shanghai, China; ^2^Institute of Health Policy, Management and Evaluation, Dalla Lana School of Public Health, University of Toronto, Toronto, ON, Canada

**Keywords:** older adults, long-term care institutions, structural characteristics, initial operating budgets, industry competition, ownership, economics

## Abstract

**Purpose:**

In the context of a rapidly aging population, this study focuses on an important issue concerning long-term care institutions, attempting to explore the relationship between structural characteristics and initial operating budgets.

**Methods:**

Data analysis was performed based on 581 long-term care institutions collected from the Shanghai Municipal Civil Affairs Bureau. Statistical analyses were conducted using IBM SPSS Statistics 24.0 with the PROCESS macro (version 3.5) for moderation analyses and Stata 16.0 for endogeneity tests.

**Results:**

The analyses revealed a significant U-shaped relationship between the gross floor areas of long-term care institutions and their initial operating budgets. Industry competition negatively moderated the effect of gross floor area on initial operating budget and also significantly exerted a direct negative effect on budgets. In addition, staffing arrangement showed a positive effect on initial operating budgets. Ownership type mattered: publicly constructed institutions had significantly lower initial operating budget compared to privately constructed institutions, whereas publicly operated institutions were associated with significantly higher initial operating budgets than privately operated ones. Institutions with leased property reported lower initial operating budgets than those with self-owned property.

**Conclusion:**

This study demonstrates that the initial operating budgets of long-term care institutions are significantly associated with their structural characteristics.

## 1 Introduction

Rapid aging of the population is a global issue (1, 2). The United Nations General Assembly put forth the “Decade of Healthy Aging” with a call to transform four areas of action (3). Providing access to long-term care for older people who need it is one of the actions. The goal is that every country should have a system of long-term care that helps people maintain relationships and take part in activities that are meaningful for them (4). The population aging trend substantially raises resource demand exemplified by long-term care services (5). The demand for care has risen and healthcare resources are one of the most critical factors contributing to a population’s health status (6).

China has 15.07 million older adult population with dementia (7), approximately 35 million disabled older adult population which is predicted to 54.72 million in 2050 (8). It is expected that by 2050, China’s older adult population will reach 400 million, and the proportion of Chinese residents aged 60 years or older is expected to be about 34.9% (9). Rapid aging of the population is a pressing concern for China (10, 11). Therefore, eldercare policies need to align with this. “Several Opinions on Accelerating the Development of the Industry of Older Adult Care Services” vigorously advocated for the construction of long-term care institutions, supporting social forces to invest long-term care institutions and operate institutions (12). “Opinions on Promoting the Development of Older Adult Care Services” stressed to promote the resolution of financing issues for long–term care institutions (13) and improve financial sustainability (14). Mitigating operational costs in healthcare are vital for ensuring financial sustainability and delivering quality care (15).

Long-term care institutions are licensed facilities that provide a range of services designed to meet the care needs of individuals with disabilities, chronic illness, or age-related conditions. Residents of long-term care institutions are mostly older adults (16). As of 2023, there were a total of 41,000 registered long-term care institutions in China (17). The majority of long-term care institutions are nonprofit organizations, including public institutions and private not-for-profit institutions. In Shanghai, public institutions assumed to provide basic older adult care services for “three-no” (those without work ability, no source of income, and no legal guardians), the rural “five-guarantee” (guaranteeing food, clothing, medical care, housing, and burial), and low-income older adults. Private care institutions reflected a market-based choice and were often complementary to public institutions (18). In the recent years, there has been an increasing number of private not-for-profit and for-profit long-term care institutions. Private institutions in total are more than public institutions in Shanghai, China. Despite the growing number and diversity of long-term care institutions in China, little attention has been paid to the financial requirements for starting and operating these facilities. Initial operating budget refers to the total amount planned by the sponsoring entity or investor during the startup phase of a long-term care institution. Understanding the initial operating budgets is crucial, as they provide the necessary resources for staffing recruitment, equipment procurement, marketing activities and facility setup, which are essential for enabling an institution to deliver quality care. Although it may seem intuitive that larger long-term care institutions require greater initial operating budgets, this relationship is not necessarily linear and lacks sufficient empirical evidence. There is a lack of applied research that has systematically examined the structural predictors of the initial operating budget for long-term care institutions. Existing healthcare literature primarily focuses on hospitals (19, 20), long-term care policies (21), and macro-level public health expenditure (22). Moreover, most studies examining operating costs have either focused on non-healthcare institutions or considered influencing factors in isolation, often overlooking the complex structural characteristics unique to long-term care institutions. Several studies have examined the U-shaped relationship between size and average costs in the non-healthcare institutions (23, 24), *yet such approaches fail to capture the interactions between multiple structural characteristics that shape budgetary requirements in long-term care institutions.* To address this gap, the present study systematically examines how structural characteristics such as gross floor area, staffing arrangement, ownership type affect initial operating budget in long-term care institutions, considering both linear and U-shaped relationships. Furthermore, it incorporates contextual moderators that have been neglected in existing studies. To provide a robust analytical basis, the study draws on insights from health, labor, property rights, and industrial economics to formulate its hypotheses. In detail, health economics highlights scale economies and cost efficiency, labor economics emphasizes human capital effects, property rights economics illustrates how ownership type shape investment incentives, and industrial economics explains how market competition influences budget planning. By combining these perspectives, the study advances a theoretical framework linking structural characteristics to initial operating budgets in long-term care institutions. Examining the financial implications of organizational structures in the context of long-term care is of great importance. On one hand, incorporating the issue of initial operating budgets into the analytical framework of economics, this study extends the field of economics to the domain of long-term care institutions. By doing so, it not only validates but also enriches the applicability of economic theories to the resource allocation challenges posed by global population aging. On the other hand, clarifying the relationships between institutional characteristics and initial operating budgets offers practical insights for policymakers, investors, and healthcare managers, enabling more informed decisions.

This manuscript is organized as follows. The conceptual framework and testable hypotheses are outlined in Section 2, while the methods used are described in Section 3. The results are presented in Section 4, while those findings and associated policy implications are discussed in Section 5. A brief concluding paragraph is in Section 6.

## 2 Conceptual framework and testable hypotheses

Conceptual frameworks help to make sense of data generating processes (DGP) and may be tailored to specific areas of inquiry in order to derive testable hypotheses. In this context, budget management theory (25, 26, 27) offers an “off-the-shelf” lens to explain and understand empirical regularities. While there are a variety of organizations associated with the provision of institutional long-term care, all managers are interested in the cost-effective provision of care. This is obvious when profit-maximization is the goal, and it is also the case when service provision is the goal as any departure from the cost-effective care provision erodes the attainment of organizational goals. Consequently, the optimization problem faced by long-term care organizations may be captured as the pursuit of organizational goals, which may include a combination of profit-maximization and service provision, subject to available resources and service provision technology. The resulting operating costs or budgets, Y, of a long-term care organization may be summarized by the following cost-function, *F* as is shown in Equation (1):

(1) Y=F(X)

Where initial operating costs depend on an array of factors, here represented by X that may include organizational gross floor area, staffing arrangement, ownership type, industry competition, and other potential covariates.

**Figure 1:**
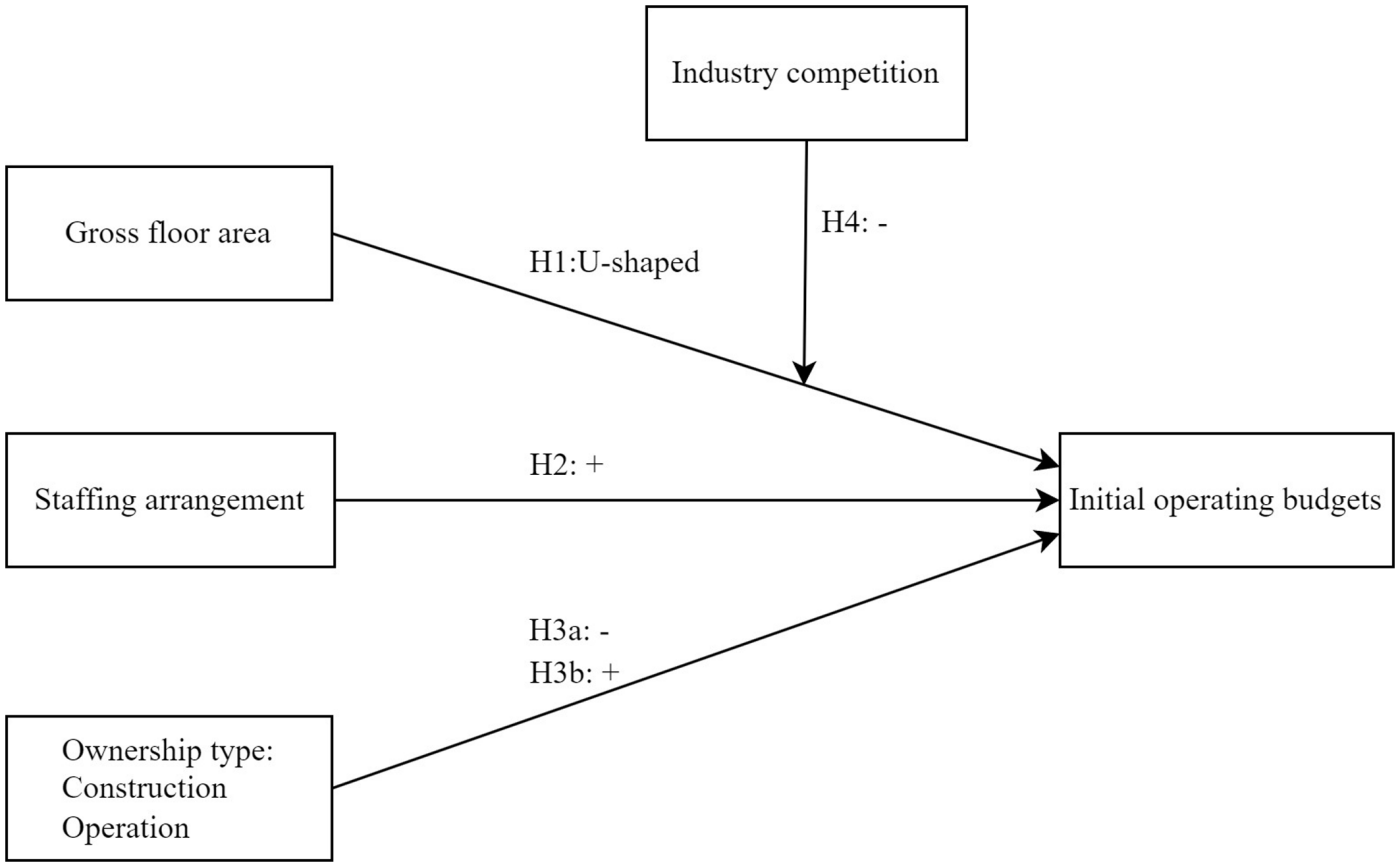
Conceptual framework.

From the perspectives of economics, this study incorporates health economics, labor economics, property rights economics and industrial economics to explain how different structural characteristics influence initial operating budgets. Structural characteristics refer to the fundamental attributes and configurations of a long-term care institution in its design and operation, including gross floor area, staffing arrangement, ownership type. Gross floor area refers to the total usable space of all floors of a building, including the interior area of each floor, used to measure the scale and capacity of a facility. Staffing arrangement in the study refers to the composition of staff in terms of professional and technical roles relative to total employees. Ownership type is the legal and organizational form of an institution’s ownership. Conceptual framework was shown in Figure 1.

### 2.1 Gross floor areas and initial operating budgets

According to health economics, the cost function of healthcare and long-term care institutions often exhibits both economies and diseconomies of scale. Because gross floor area can have an effect on operating cost (28) and initial operating budget, it is recognized as one of the important factors for cost estimating (29). As the size increased, the unit production cost initially declined, reaching a critical threshold before rising again (30). For long-term care institutions with relatively small gross floor areas, although their overall scale is limited, they must still include all essential service facilities and functional zones. As the gross floor areas increases, long-term care institutions may achieve temporary cost savings due to the distribution of fixed costs over a larger area, reflecting economies of scale. However, when managerial ability does not increase in tandem with institutional size, diseconomies of scale may emerge (31). This leads to a significant rise in investment because of increased complexity and bureaucratic inefficiencies. In this sense, relatively modest-sized organizations can achieve a major portion of the possible cost savings associated with size (23, 24), whereas very small or very large institutions are less efficient. Thus, institution with moderate gross floor area are able to realize economies of scale by spreading fixed costs more efficiently, whereas very small or excessively large size encounter diseconomies of scale due to underutilization of resources or increased managerial complexity. We suppose that:

H1: The relationship between the gross floor area of long-term care institutions and their initial operating budgets is non-linear, U-shaped.

### 2.2 Staffing arrangement and initial operating budgets

From labor economics, skilled professionals are a critical input in long-term care production functions. Human capital is represented by people who are carriers of knowledge, ideas and experience that contribute to increasing the performance and competitiveness of the institution and the entire society (32). Professional and technical staff represents the core human capital which is associated with better outcomes (33), commanding higher wage premiums and requiring longer education (34). A higher proportion of professional and technical staff to total employees will lead to the higher expenditures on recruitment, training and compensation. Additionally, a greater share of professional and technical staff reflects a higher level of service specialization, which usually correlates with more sophisticated infrastructure. Therefore, a higher proportion of professional and technical staff has more labor capital requirements, leading to the following hypothesis:

H2: The relationship between staffing arrangement of long-term care institutions and their initial operating budgets is significantly positive.

### 2.3 Ownership type and initial operating budgets

From economics of property rights, ownership type reflects varying methods of resource acquisition and institutional constraints, thereby impacting an organization’s operating costs (35). Under conditions of clear property rights and profit-driven incentives, privately constructed and operated long-term care institutions have a strong incentive to avoid high-cost groups who are regarded as costly (36). Privately constructed and operated care institutions prioritize cost control through staffing and material cost reductions (37). Private institutions often allow for more flexible spending and fewer regulatory burdens. Publicly constructed long-term care institutions often benefit from more government support, leading to lower initial operating budgets compared to their privately constructed counterparts. Publicly operated institutions tend to bear higher initial operating costs due to strict regulations (38, 39) and multiple government-mandated functional tasks such as caring for low-income groups. Public institutions tend to have higher operational costs and be less efficient (40, 41), while private facilities often operate more efficiently, providing lower-cost services than publicly run ones (42). Therefore, the following hypotheses are proposed:

H3: The relationship between ownership type of long-term care institutions and their initial operating budgets is significant.

H3a: Publicly constructed long-term care institutions have lower initial operating budgets than privately constructed institutions.

H3b: Publicly operated long-term care institutions have higher initial operating budgets than privately operated counterparts.

### 2.4 Moderating role of industry competition

Industry competition refers to the extent to which different institutions within an industry compete for customers, resources, and market share. From industrial economics, industry competition shapes the investment decisions (43) and limited competition can lead to overinvestment. In the context of long-term care, industry competition exerts external pressure for institutions to operate efficiently and control expenses (44). In less competitive environments, institutions face weaker external pressure to control costs and may engage in “luxury” investments such as larger gross floor area, higher-end facilities. As a result, this behavior leads to a stronger relationship between gross floor area and initial operating budget. By contrast, in highly competitive environments, long-term care institutions are pressured to control costs. It leads to a reduction in space-related expenses. In the meantime, productivity of staff or the use of technological support enable these long-term care institutions to operate more efficiently, which mitigates the effect of gross floor areas on the operating budget. Therefore, if the industry competition becomes more intense, the effect of gross floor area on the initial operating budget is weaker, we propose:

H4: The moderating effect of industry competition on the relationship between the gross floor area and initial operating budgets in long-term care institutions is significantly negative.

## 3 Methods

### 3.1 Data sources and study size

Data were obtained from the Shanghai Municipal Civil Affairs Bureau in 2020 that recorded the characteristics of long-term care institutions, such as initial operating budget, gross floor area etc. While the database included 753 long-term care institutions.

Considering that COVID-19 pandemic likely impacted the operations of long-term care institutions through factors such as changes in procurement costs and operational adjustments, we deleted 40 long-term care institutions established after 2019.

An additional 111 long-term care institutions were excluded due to missing important data like gross floor area, initial operating budget etc. Among these, 57 long-term care institutions have been closed. 4 long-term care institutions were ready to close. 1 long-term care institution was in the process of obtaining licenses. 28 long-term care institutions were suspended. 21 long-term care institutions were with incomplete filings during their registration process.

In addition, 21 long-term care institutions were deleted due to data not conforming to common sense.

Thus, the resulting analysis sample comprises 581 institutions. We recognized that the exclusion of some institutions might introduce sample selection bias. The Mann–Whitney U test revealed that the distributions of gross floor area, initial operating budget, ownership type and geographic location showed no significant differences. This suggested that the excluded institutions were not systematically different from the retained sample. However, to further control for potential selection bias, we followed the approach of Pinelli et al. (45) and applied the Heckman procedure. To ensure the identification, we included exclusion restrictions such as closure status, suspension status and incomplete registration in the selection equation. We computed the residuals and used them to calculate the inverse Mills ration included as an additional control variable in our empirical tests.

### 3.2 Variables and operational definition

#### 3.2.1 Dependent variables

As a continuous variable, initial operating budgets are measured as the total amount of funds planned by the sponsoring entity or investor for each institution’s initial stage of operation. Before the institution begins providing services, initial operating budget is filed with the relevant government authorities.

#### 3.2.2 Independent variables

Gross floor area is measured in square meters, usually obtained from government registration records. Staffing arrangement is measured as proportion of professional and technical staff to total employees. Ownership type is a nominal variable, taking reference from some scholars (46). Ownership type is operationalized through two binary dimensions: Construction and Operation. Let Construction be 1 if the long-term care institution is publicly constructed and 0 otherwise. Let Operation be 1 if the long-term care institution is publicly operated and 0 otherwise.

#### 3.2.3 Moderating variable

Industry competition functions as an external factor in the market. It is measured by the number of long-term care institutions per 10,000 registered permanent residents aged 60 and above when the institutions established.

#### 3.2.4 Control variables

Nature of service premises is a nominal variable. We take on the value of 1 for service premise nature as leased property, 0 for self-owned property and other. Geographic location, following Mano and Rosenberg (47), is a binary variable that takes on the value of 1 for institutions located in the urban, and 0 otherwise. The type of service is measured by a binary variable that takes on the value of 1 for care for persons with severe disabilities and/or those with dementia, and 0 for other.

#### 3.2.5 Instrumental variables

Land type refers to the officially designated land-use category such as industrial, commercial, public green land, or others as recorded in urban planning and land administration documents. We employ two dummy variables: Landtype_ICL and Landtype_PGL. If the long-term care institution is located on industrial or commercial land Landtype_ICL is 1; if not, it is 0. Let Landtype_PGL be 1 if the long-term care institution is in public green land and 0 otherwise.

### 3.3 Econometric model

The determinants of the initial operating budget in the baseline models are expressed as Equations (2–4):

(2) $$Y=\beta_{0}+\beta_{1}X_{1}+\beta_{2}X_{2}+\beta_{3}X_{3}+\beta_{4}X_{4}+\varepsilon$$

(3) $$Y=\beta_{0}+\beta_{1}X_{1}+\beta_{2}X_{2}+\beta_{3}X_{3}+\beta_{4}X_{4}+\beta_{5}X_{1}^{2}+\varepsilon$$

(4) $$Y=\beta_{0}+\beta_{1}X_{1}+\beta_{2}X_{2}+\beta_{3}X_{3}+\beta_{4}X_{4}+\beta_{5}X_{1}^{2}+\sum_{d=1}^{3}\mu_{d}Z_{d}+\varepsilon$$

Here, the dependent variable represents the initial operating budget. X_1_ is the first independent variable, gross floor area. X_2_ is the second independent variable, staffing arrangement. X_3_ and X_4_ are the third and fourth independent variable, respectively. Z_d_ is the d^th^ control variable, and β_0_ represents the intercept; β_1_, β_2_, β_3_ and β_4_ are the regression coefficient on the first, second, third and fourth independent variable, respectively. β_5_ is the regression coefficient on the square of first independent variable. $\mu_{d}$ is the regression coefficient on the d^th^ control variable. There are three control variables. And ε represents the error term. Positive regression coefficient value of an independent variable indicates a positive influence of an independent variable on the dependent variable, vice versa.

To account for the moderating effect of variable M, the models are specified according to Equations (5–7):

(5) $$\begin{array}{l} Y=\beta_{0}+\beta_{1}X_{1}+\beta_{2}X_{2}+\beta_{3}X_{3}+\beta_{4}X_{4}+\\ \beta_{5}M+\rho_{1}(X_{1}\times M)+\sum_{d=1}^{3}\mu_{d}Z_{d}+\varepsilon \end{array}$$

(6) $$\begin{array}{l} Y=\beta_{0}+\beta_{1}X_{1}+\beta_{2}X_{2}+\beta_{3}X_{3}+\beta_{4}X_{4}+\beta_{5}M+\\ \rho_{1}(X_{1}\times M)+\sum_{d=1}^{3}\mu_{d}Z_{d}+\beta_{6}X_{1}^{2}+\varepsilon \end{array}$$

(7) $$\begin{array}{l} Y=\beta_{0}+\beta_{1}X_{1}+\beta_{2}X_{2}+\beta_{3}X_{3}+\beta_{4}X_{4}+\beta_{5}M+\rho_{1}(X_{1}\times M)+\\ \sum_{d=1}^{3}\mu_{d}Z_{d}+\beta_{6}X_{1}^{2}+\rho_{2}(X_{1}^{2}\times M)+\varepsilon \end{array}$$

Where ρ_1_ is the regression coefficient, capturing the extent to which the effect X_1_ on Y varies depending on the level of M. A significant of regression coefficient indicates that M moderates the relationship between X_1_ and Y. ρ_2_ represents how much the quadratic effect of X_1_ on Y is influenced by M.

### 3.4 Variable processing and data analysis

#### 3.4.1 Variable processing

Skewness and kurtosis of variables were examined to assess normality of the key variables. Variables like initial operating budget, gross floor area, and staffing arrangement exhibited significant skewnness and/or kurtosis. Many regression analyses involve transformation of variables (48). Therefore, for variables with significant non-normality, a natural log transformation was applied to improve distributional properties. After processing, the skewness and kurtosis of the variables returned to acceptable ranges (skewness ≤ 2 and kurtosis ≤ 7). It was shown in Table 1.

**Table 1:** Skewness, kurtosis, and transformation of variables.

Variable	Original skewness	Original kurtosis	Transformation applied	New skewness	New kurtosis
Initial operating budget	11	165	Ln(IOB)	0.877	0.238
Gross floor area	5.585	60.022	LnGFA_centered	0.189	−0.118
Staffing arrangement	1.963	7.692	Ln(SA + 1)_centered	1.435	4.346
Untransformed variables					
Ownership type_Construction	0.254	−1.942	-	-	-
Ownership type_Operation	0.933	−1.134	-	-	-
Industry competition	−0.197	−0.607	-	-	-
Type of service	1.108	2.454	-	-	-
Nature of service premises	−0.649	−1.584	-	-	-
Geographic location	0.079	−2.001	-	-	-

Gross floor area was mean-centered by first applying the natural logarithm to the original variable GFA and then subtracting the mean of the logged values, resulting in the centered logarithmic variable LnGFA_centered. To handle zero values, staffing arrangement was log-transformed using the natural logarithm after adding one. Ln (SA+1)_centered represents Ln(SA+1) minus the mean of Ln(SA+1).

#### 3.4.2 Data analysis

Statistical analyses were conducted with IBM SPSS Statistics 24.0, employing the PROCESS macro (version 3.5) to rigorously examine moderation effects. Descriptive analyses were pursued. Categorical variables were expressed as frequencies and percentages (49). As several variables, including continuous, and nominal variables were used in our study, we conducted Spearman’s rank correlation for continuous variables. Spearman’s rank correlation coefficients between variables were calculated based on the transformed variables to ensure consistency with the subsequent regression models. Point-Biserial correlation for a binary variable and a continuous variable, and Phi correlation analysis between a binary variable and a binary variable were conducted. In the linear regression analysis, tolerance and the variance inflation factor (VIF) measured potential multicollinearity. To evaluate the suitability of the regression model, graphical diagnostics of the residuals were performed. We first examined influence of independent variables on the dependent variable. Then we added control variables together with independent variables to estimate the model using ordinary least squares (OLS) regression. Afterwards, we assessed the moderating effect of industry competition, employing the PROCESS macro developed by Hayes. It can offer added advantages such as Johnson-Neyman analysis, and improved the robustness and interpretability of the results (50). Paying attention to robustness checks could contribute to reducing bias and increasing reliability (51). We conducted robustness checks using additional control variables, bootstrap resampling, and winsorization. Finally, we employ the instrumental variable method to address possible endogeneity issues, considering that endogeneity was a critical concern in research methodologies (52).

## 4 Results

### 4.1 Descriptive statistics

The results of the descriptive statistical analysis of the variables were shown in Table 2.

**Table 2:** Descriptive statistics of variables.

Variable	Type	Mean	S. D.	Minimum	Maximum
Dependent variable					
Initial operating budget (CNY)	Continuous	1,350,350	493	10,000	86,500,000
Independent variables					
Gross floor area (square meters)	Continuous	6,023	6,934	360	100,000
Staffing arrangement (%)	Continuous	10	9	0	67
Ownership type_Construction	Binary	The frequency of privately constructed institution is 327 (56.1% of the sample). The frequency of publicly constructed institution is 254 (43.6% of the sample).			
Ownership type_Operation	Binary	The frequency of privately operated institution is 413 (70.8% of the sample). The frequency of publicly operated institution is 168 (28.8% of the sample).			
Moderating variables					
Industry competition	Continuous	0.744	0.284	0.007	1.121
Control variables					
Type of service	Binary	The frequency of care provided for persons with severe disabilities and/or dementia is 80 (13.7%). The frequency of care provided for persons without severe disabilities and those without dementia is 501(85.9%).			
Nature of service premises	Binary	The frequency of institution with leased service premises is 380 (65.4%). The frequency of institution with owned property and others is 201 (34.6%).			
Geographic location	Binary	The frequency of institution situated in urban area is 279 (47.9%). The frequency of institution situated in suburban area is 302 (51.8%).			
Instrumental variables					
Land type_ICL	Binary	The frequency of institution located on industrial or commercial land is 12 (2.1% of the sample). The frequency of institution which is not located on industrial or commercial land is 569 (97.9%).			
Land type_PGL	Binary	The frequency of institution located on public green land is 14 (2.4%). The frequency of institution which is not located on public green land is 567 (97.6%).			

*N* = 581.

### 4.2 Correlation analyses

Spearman’s rank correlation analysis between continuous variables was shown in Table 3. Point-Biserial correlation between binary and continuous variables was shown in Table 4. The result of Phi coefficient between a binary variable and a binary variable was shown in Table 5.

**Table 3:** Spearman’s rank correlation analysis between continuous variables.

Variable	1	2	3	4
1 Ln(IOB)	1			
2 LnGFA_centered	0.255**	1		
3 Ln(SA + 1)_centered	0.152**	0.166**	1	
4 Industry competition	−0.077**	0.087**	0.071**	1

**Correlation is significant at the 0.05 level (one-tailed). LnGFA_centered represents Ln(GFA) minus the mean of Ln(GFA). LnGFA_c_squared represents a squared term of the centered log-transformed gross floor area.

**Table 4:** Point-Biserial correlation between a binary variable and a continuous variable.

Variable	Initial operating budget	Gross floor area	Staffing arrangement	Industry competition
Type of service	0.034	0.150**	0.018	−0.016
Ownership type_Construction	0.090**	0.084**	−0.081**	−0.059
Ownership type_Operation	0.136**	0.021	−0.062	−0.112**
Geographic Location	−0.071**	−0.242**	0.064	0.075**
Nature of service premises	−0.168**	−0.179**	0.059	0.130**
Land type_ICL	0.028	0.235**	−0.042	−0.021
Land type_PGL	0.013	0.174**	0.009	−0.003

**Correlation is significant at the 0.05 level (one-tailed).

**Table 5:** The result of Phi coefficient between a binary variable and a binary variable.

Variable	1	2	3	4	5	6	7
1. Type of service	1						
2. Ownership type_Construction	−0.020	1					
3. Ownership type_Operation	−0.057	0.624**	1				
4. Geographic Location	−0.094**	−0.166**	−0.172**	1			
5. Nature of service premises	−0.024	−0.322**	−0.310**	0.257**	1		
6. Land type_ICL	−0.023	−0.104**	−0.093**	−0.042	0.004	1	
7. Land type_PGL	0.083**	−0.055	−0.066	0.042	−0.029	−0.021	1

**.Correlation is significant at the 0.05 level.

### 4.3 Benchmark regression analyses

In Table 6, Column 1 shows the regression outcomes of the dependent variable on independent variables only. Column 2 shows the regression outcomes of the dependent variable on independent variables and LnGFA_c_squared variable, and column 3 controls for variables based on column 2.

**Table 6:** Benchmark regression analysis results.

Variable	M1	M2	M3
Constant	3.406^**^	3.274^**^	3.492^**^
LnGFA_centered	0.518^**^	0.492^**^	0.523^**^
Ln(SA + 1)_centered	2.618^**^	2.510^**^	2.470^**^
Ownership type_Construction	−0.429^**^	−0.474^**^	−0.527^**^
Ownership type_Operation	0.530^**^	0.565^**^	0.508^**^
LnGFA_c_squared	-	0.191^**^	0.172^**^
Type of service	-	-	−0.174
Nature of service premises	-	-	−0.380^**^
Geographic location	-	-	0.222^*^
*R* ^2^	0.121	0.136	0.153
Adjusted *R*^2^	0.115	0.129	0.141
*F* value	19.785^**^	18.176^**^	12.848^**^

Dependent variable is Ln (IOB). *Denote being significant at 0.1 level. **Denote being significant at 0.05 level.

Results of Model 1 showed that the regression model was significant, with an F-test value of 19.785 and *p*-value < 0.01. An R-squared value of 0.121 was obtained for the model. The regression coefficient for LnGFA_centered (ß = 0.518, *p* < 0.05) indicated that, holding other factors constant, gross floor area had a significant and positive relationship with the initial operating budget. The regression coefficient for Ln (SA + 1)_centered (ß = 2.618, *p* < 0.05) indicated that, staffing arrangement positively and significantly impacted initial operating budgets. H2 was confirmed. The regression coefficient for Ownership type_Construction (ß = −0.429, *p* < 0.05) indicated that, holding other factors constant, publicly constructed institutions had significantly lower initial operation budgets than privately constructed institutions. H3a was supported. The coefficient for Ownership type_Operation (ß = 0.530, *p* < 0.05) suggested that publicly operated institutions had significantly higher initial operation budgets than privately operated ones. H3b was supported. Taken together, H3 was supported.

Results of Model 2 showed that the regression model was significant, with an F-test value of 18.176 and *p*-value < 0.01. An R-squared value of 0.136 was obtained. The regression coefficient for LnGFA_centered (ß = 0.492, *p* < 0.05) was positive and significant. Similarly, staffing arrangements had a strong positive effect (β = 2.510, *p* < 0.01). Ownership type_Construction was negatively related to Ln(IOB) (β = −0.474, *p* < 0.01), whereas Ownership type_Operation showed a positive effect (β = 0.565, *p* < 0.01). The coefficient for LnGFA_c_squared was positive and significant (β = 0.191, *p* < 0.01). Therefore, H1 was supported, holding other factors constant, gross floor area had a U-shaped relationship with the initial operating budget. H2, H3, H3a and H3b were all supported.

Model 3 incorporates additional control variables, including type of service, nature of service premises, and geographic location. The model explains 15.3% of the variance in Ln(IOB) with an F-test value of 12.848 and *p*-value < 0.01. The additional controls provide incremental explanatory power. The main effects of LnGFA_centered (β = 0.523, *p* < 0.01) and staffing arrangements (β = 2.470, *p* < 0.01) remain positive. The main effects of Ownership type_Construction (β = −0.527, *p* < 0.01) and Ownership type_Operation (β = 0.508, *p* < 0.01) remain robust too. Type of service had no significant impact on the initial operating budget (ß = −0.174, *p* > 0.05). Nature of service premises is negatively and significantly associated with Ln(IOB) (β = −0.380, *p* < 0.05), while geographic location is positively significant (β = 0.222, *p* < 0.1). The coefficient for LnGFA_c_squared was positive and significant (β = 0.172, *p* < 0.01). Therefore, H1, H2, H3, H3a and H3b were further supported.

### 4.4 Moderation analyses

PROCESS macro for SPSS (Model 1) was used to test moderation effect. Table 7 showed results. Column 1 shows the regression outcomes of the dependent variable on independent variables and control variables. Column 2 shows the regression outcomes of the dependent variable on LnGFA_c_squared variable based on column 1, and column 3 adds LG_csi based on column 2. Whatever in Model 4 (ß = −0.563, *p* < 0.05), Model 5 (ß = −0.584, *p* < 0.05) or Model 6 (ß = −6.000, *p* < 0.05), the regression coefficient for the interaction between LnGFA_centered and industry competition were all negative and significant. The regression coefficients for LnGFA_centered and LnG_cs were significant and positive. The interaction between LnGFA_c_squared and industry competition in Model 6 is not significant (ß = −0.234, *p* > 0.05). The two interaction terms captures how external competitive pressure constrains additional spending in larger institutions, aligning with the economic logic that competition incentivizes cost control at higher scales without altering the fundamental scale economies and diseconomies that generate the U-shaped pattern. This suggested that industry competition altered the marginal effect of gross floor area on initial operating budget, particularly in the upper (right) half of the U-shaped curve, but did not affect the overall curvature of the relationship. These results provided some empirical support for H4.

**Table 7:** Results with PROCESS macro.

Variable	Abbreviation	M4	M5	M6
Constant	-	4.072^**^	3.925^**^	3.811^**^
LnGFA_centered	LnGFA_c	0.985^**^	0.973^**^	0.990^**^
Ln(SA + 1)_centered	LnSA_c	2.650^**^	2.567^**^	2.534^**^
Construction	Constru	−0.457^**^	−0.491^**^	−0.496^**^
Operation	Opera	0.401^**^	0.433^**^	0.439^**^
Industry competition	InduC	−0.624^**^	−0.595^**^	−0.430
LnGFA_centered×Industry competition	Int_1	−0.563^**^	−0.584^**^	−0.600^**^
LnGFA_c_squared	LnG_cs	-	0.168^**^	0.333^**^
LnGFA_c_square×Industry competition	LG_csi	-	-	−0.234
Type of service	ServiT	−0.214	−0.218	−0.225
Nature of service premises	ServiP	−0.379^**^	−0.343^**^	−0.348^**^
Geographic location	GL	0.279^**^	0.246^*^	0.245^*^
*R* ^2^	-	0.157	0.169	0.171
MSE	-	1.993	1.968	1.968
*F* value	-	11.807^**^	11.578^**^	11.000^**^

Dependent variable is Ln (IOB). Because the PROCESS macro demands for the variable length, we use abbreviation of variables when using PROCESS macro. *Denote being significant at 0.1 level. **Denote being significant at 0.05 level.

Tables 8–10 presented the conditional effects of log-transformed and mean-centered gross floor area (LnGFA-c) on the log of initial operating budget (LnIOB) at varying levels of industry competition. They were in accordance with Model 4, Model 5,and Model 6, respectively. The results indicated that the effect of LnGFA-c on LnIOB decreased as the level of industry competition increased. Specifically, at a low level of industry competition (value = 0.393), the effect was strong and significant. At the mean level of industry competition (value = 0.712), the effect remained significant but was slightly weaker. At a high level of industry competition (value = 1.121), the effect further decreased, though it remained statistically significant. The Johnson-Neyman technique revealed no statistical transition points within the observed range of industry competition. This suggested that the effect of gross floor area on initial operating budget remained statistically significant across all levels of competition observed in the sample, indicating a consistent moderating effect.

**Table 8:** Conditional effects of focal predictor on dependent variable at varying levels of industry competition without LnGFA_c_squared as a covariate.

Industry competition	Effect	se	*t*	*p*	LLCI	ULCI
0.393	0.764	0.115	6.665	0.000	0.539	0.989
0.712	0.584	0.077	7.604	0.000	0.433	0.735
1.121	0.354	0.123	2.866	0.004	0.111	0.596

Industry competition values in conditional tables are the 16th, 50th, and 84th percentiles. Level of confidence for all confidence intervals in output: 95.000. There are no statistical significance transition points within the observed range of the moderator found using the Johnson-Neyman method.

**Table 9:** Conditional effects of focal predictor on dependent variable at varying levels of industry competition.

Industry competition	Effect	se	*t*	*p*	LLCI	ULCI
0.393	0.744	0.114	6.519	0.000	0.517	0.968
0.712	0.557	0.077	7.249	0.000	0.406	0.708
1.121	0.318	0.123	2.584	0.010	0.076	0.561

Industry competition values in conditional tables are the 16th, 50th, and 84th percentiles. Level of confidence for all confidence intervals in output: 95.000. There are no statistical significance transition points within the observed range of the moderator found using the Johnson-Neyman method.

**Table 10:** Conditional effects of focal predictor on dependent variable at varying levels of industry competition, with LnGFA_c_square × Industry Competition (LG_csi) as a covariate.

Industry competition	Effect	se	*t*	*p*	LLCI	ULCI
0.393	0.754	0.115	6.586	0.000	0.529	0.979
0.712	0.563	0.077	7.302	0.000	0.411	0.714
1.121	0.317	0.123	2.571	0.010	0.075	0.559

Industry competition values in conditional tables are the 16th, 50th, and 84th percentiles. Level of confidence for all confidence intervals in output: 95.000. There are no statistical significance transition points within the observed range of the moderator found using the Johnson-Neyman method.

### 4.5 Diagnostic checks for benchmark regression models

**Figure 2:**
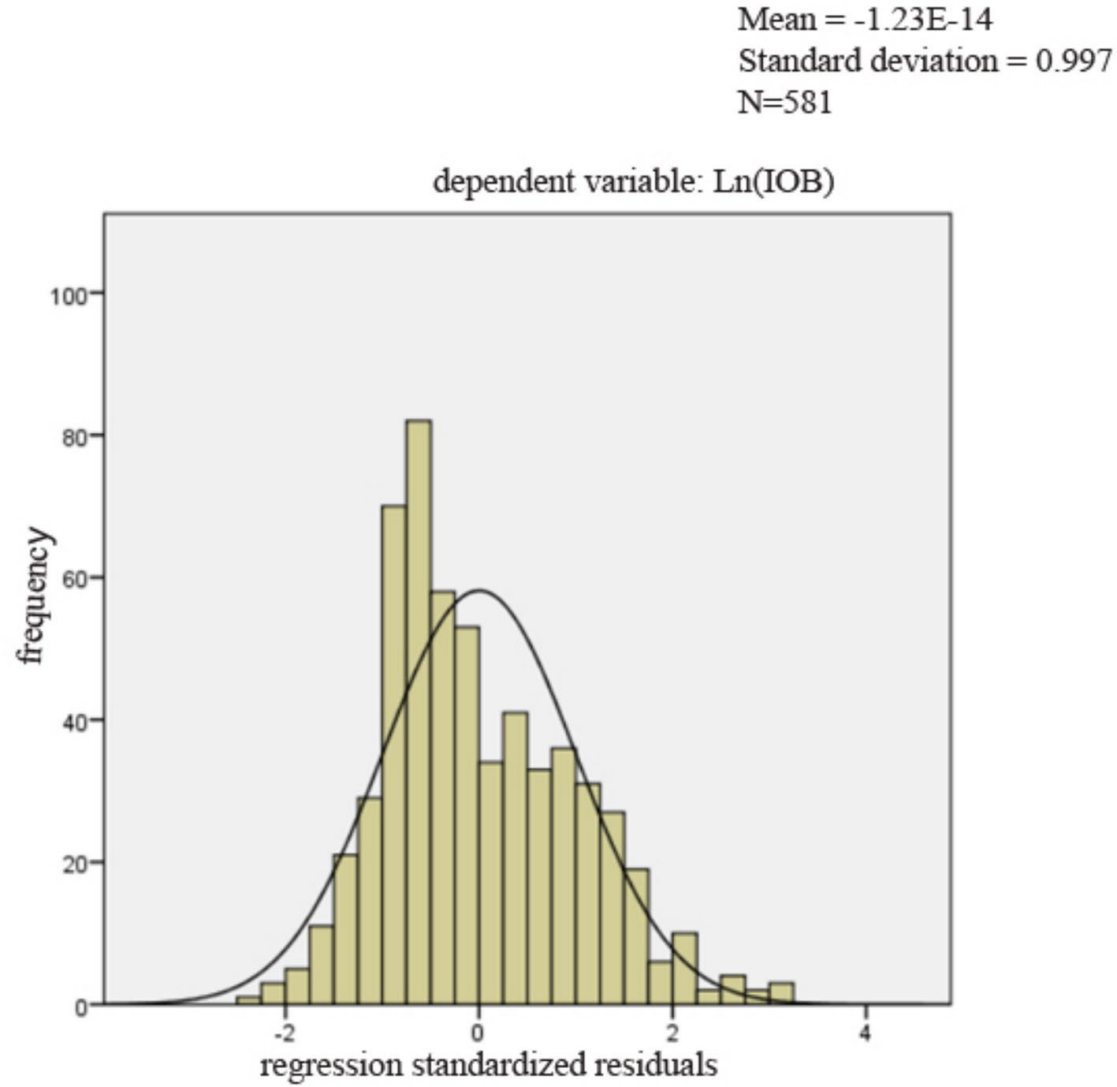
The histogram of regression standardized residuals.

**Figure 3:**
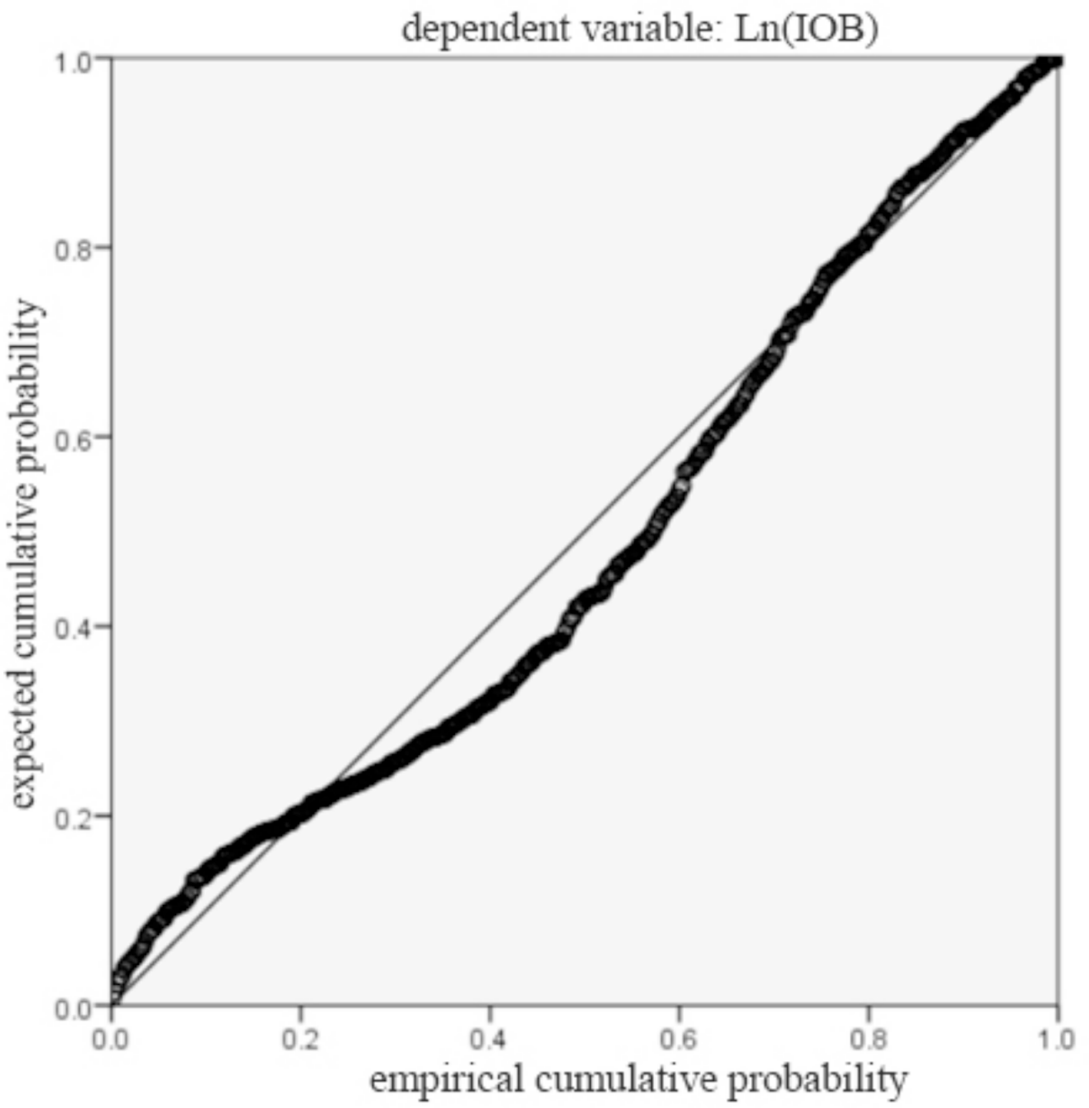
The normal P–P plot of standardized residuals.

**Figure 4:**
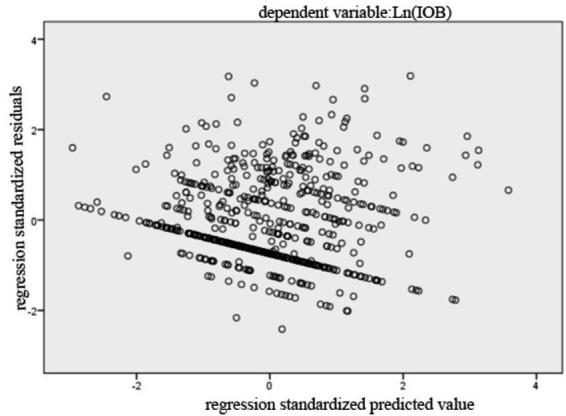
The scatter plot.

Table 11 presented the diagnostic results for Model 1 to Model 3. The results iinndicated no multicollinearity, as all VIF values were below 10 (53). Figure 2 presented the histogram of regression standardized residuals for the Model 1. The residuals were approximately normally distributed, as evidenced by the bell-shaped curve that aligns well with the fitted normal distribution line. The mean of the standardized residuals was close to zero, and the standard deviation is close to one, satisfying the assumption of normality in the residuals. Therefore, the normality assumption of the linear regression model was reasonably met. Figure 3 showed that the points were approximately aligned along the diagonal line. Figure 4 displayed the scatter plot of standardized residuals against standardized predicted values, where residuals were randomly scattered around zero with no apparent signs of heteroscedasticity or nonlinearity, suggesting good homoscedasticity and linear fit of the model. Results for Model 2 and Model 3 were similar and are available upon request. Therefore, the benchmark regression analyses met the Gauss-Markov conditions and the ordinary least squares (OLS) estimates can be considered unbiased and efficient.

**Table 11:** Results of tolerance and variance inflation factor (VIF) in the regression.

Variable	Tolerance in M1	VIF in M1	Tolerance in M2	VIF in M2	Tolerance in M3	VIF in M3
Constant	-	-	-	-	-	-
LnGFA_centered	0.958	1.044	0.946	1.057	0.790	1.265
Ln(SA + 1)_centered	0.962	1.039	0.960	1.041	0.943	1.060
Ownership type_Construction	0.469	2.134	0.466	2.147	0.460	2.175
Ownership type_Operation	0.474	2.109	0.473	2.116	0.461	2.169
LnGFA_c_squared	-	-	0.975	1.025	0.961	1.040
Type of service		-	-	-	0.951	1.052
Nature of service premises		-	-	-	0.828	1.208
Geographic location		-	-	-	0.812	1.231

Dependent variable is Ln (IOB).

### 4.6 Robust analyses

The above results provide evidence that H1, H2, H3, H3a, and H3b were supported totally. H4 was given only some empirical support. However, further examinations were done to validate these findings.

First, we test the robustness of our results to additional control variables such as degree of population aging, Shanghai GDP in the year of establishment, CPI in the year of establishment. Despite the inclusion in the model of additional control variables, it can be seen from Model 7 to Model 9 in Table 12 that H1, H2, H3, H3a and H3b were still supported, demonstrating the robustness of the models. Second, we employed the bootstrap resampling method with 5,000 iterations (Mersenne Twister; seed = 20,250,826) to re-estimate the standard errors and confidence intervals. The results shown in Table 13 remained consistent with the baseline regression, confirming the robustness of our findings. Besides, we found that despite the inclusion in the model of variable such as degree of population aging, Shanghai GDP in the year of establishment, the regression coefficient for the interaction between LnGFA_centered and industry competition were all negative and significant while the interaction between LnGFA_c_squared and industry competition was not significant. H4 was supported to some degree. Third, to mitigate the influence of outliers, we conducted winsorization at the 1st and 99th percentiles of the variables and re-estimated the regression models. The results are not shown here, but they can be provided upon request. Taken together, the results remained consistent with the baseline regression, suggesting that our findings are robust.

**Table 12:** Regression analysis results with additional control variables.

Variable	M7	M8	M9
Constant	3.860^**^	4.190^**^	4.223^**^
LnGFA_centered	0.521^**^	0.545^**^	0.546^**^
Ln(SA + 1)_centered	2.436^**^	2.504^**^	2.487^**^
Ownership type_Construction	−0.536^**^	−0.494^**^	−0.500^**^
Ownership type_Operation	0.519^**^	0.476^**^	0.475^**^
LnGFA_c_squared	0.172^**^	0.174^**^	0.173^**^
Type of service	−0.170	−0.179	−0.177
Nature of service premises	−0.376^**^	−0.338^**^	−0.337^**^
Geographic location	0.270^*^	0.305^*^	0.309^*^
Degree of population aging	−0.011	−0.014	−0.015
GDP of Shanghai in the establishing year		−1.113E-5^**^	−1.018E-5^**^
CPI of Shanghai in the establishing year			−0.022
*R* ^2^	0.153	0.162	0.162
Adjusted *R*^2^	0.140	0.147	0.146
*F* value	11.433^**^	10.980^**^	9.986^**^

Dependent variable is Ln (IOB). *Denote being significant at 0.1 level. **Denote being significant at 0.05 level.

**Table 13:** Regression analysis results using bootstrap resampling method.

Variable	B	Bias	Standard error	Significance (two-tailed)	LLCI	ULCI
Constant	3.492^**^	0.001	0.154	0.000	3.185	3.793
LnGFA_centered	0.523^**^	−0.001	0.077	0.000	0.371	0.671
Ln(SA + 1)_centered	2.470^**^	0.002	0.890	0.005	0.698	4.223
Ownership type_Construction	−0.527^**^	−0.003	0.168	0.003	−0.848	−0.195
Ownership type_Operation	0.508^**^	0.004	0.204	0.014	0.110	0.909
LnGFA_c_squared	0.172^**^	−0.002	0.062	0.005	0.041	0.286
Type of service	−0.174	0.004	0.182	0.337	−0.527	0.182
Nature of service premises	−0.380^**^	−0.001	0.141	0.008	−0.663	−0.117
Geographic location	0.222	0.001	0.137	0.102	−0.049	0.488
*R* ^2^	0.153	-	-	-	-	-
Adjusted *R*^2^	0.141	-	-	-	-	-
*F* value	12.848^**^					

Dependent variable is Ln (IOB). Unless otherwise specified, the bootstrap results are based on 5,000 bootstrap samples. *Denote being significant at 0.1 level. **Denote being significant at 0.05 level.

### 4.7 Endogeneity tests

The results were shown in Table 14. In the first-stage regression, The *F*-value was 22.040. The minimum eigenvalue statistic was 18.390, exceeding the Stock-Yogo critical value for 10% maximal relative bias. Thereby, the IVs were sufficiently strong. Land type_ICL (ß = 1.024, *p* < 0.05) and Land type_PGL (ß = 0.884, *p* < 0.1) were significantly linked to LnGFA, further supporting instrument relevance. In the second-stage regression, the relationship between gross floor area and initial operating budgets remained significant (ß = 0.702, *p* < 0.05). Moreover, the Durbin and Wu–Hausman tests indicated that LnGFA was not significantly endogenous and the overidentification test confirmed the validity of the instruments.

**Table 14:** Results of the two-stage regression.

Variable	Ln(GFA)	Ln(IOB)
Constant	8.321^**^	−1.855
Ln(SA + 1)_centered	2.319^**^	2.267^**^
Ownership type_Construction	0.209^**^	−0.499^**^
Ownership type_Operation	−0.194^*^	0.475^**^
Industry competition	0.325^**^	−0.586^**^
Type of service	0.414^**^	−0.245
Nature of service premises	−0.194^**^	−0.360^**^
Geographic location	−0.524^**^	0.352^*^
Land type_ICL (IV)	1.024^**^	-
Land type_PGL (IV)	0.884^**^	-
Predicted Ln(GFA)	-	0.702^**^
*R* ^2^	0.258	0.146
Adjusted *R*^2^	0.246	-
Root MSE	0.747	1.409
number of observation	581	581
Wald chi 2 (8)	-	51.250
*F* value	22.040^**^	-

*Denote being significant at 0.1 level. **Denote being significant at 0.05 level.

## 5 Discussion

This study investigated the relationship between structural characteristics of long-term care institutions and their initial operating budgets in the context of a rapidly aging population. Our findings provide novel evidence that structural factors—including gross floor area, staffing arrangements, ownership type—are significantly associated with the financial requirements of establishing long-term care institutions. Although some independent variables showed statistically significant coefficients, the overall R-squared value was modest. This was not uncommon in empirical research dealing with complex human behavior and organizational phenomena. Some published papers (54, 55) in leading journals report R-squared values below 0.2, yet are considered valuable for the causal insights they provide. A low R-squared did not invalidate the usefulness of a model, especially when the estimated coefficients were statistically significant and theoretically meaningful (56). Ozili argues that a model with an R-squared that was between 0.10 and 0.50 was good provided that some or most of the explanatory variables are statistically significant (57). In our study, despite the moderate explanatory power, the estimated coefficients are statistically significant, and aligned with theoretical expectations.

The observed relationship is consistent with causal interpretation. During the preparation phase, structural characteristics of a long-term care institution such as gross floor area are typically determined by construction standards and policy requirements. The initial operating budget is then planned to meet these predefined conditions and reported to relevant authorities, indicating that structural characteristics drive the budget, rather than the reverse.

### 5.1 Main findings and literature comparisons

First, this study confirmed a significant U-shaped relationship between gross floor area and the initial operating budget, suggesting that economies of scale coexist with diseconomies of scale. From the perspective of health economics, smaller institutions may incur disproportionately higher per-unit costs due to fixed investment requirements, while very large institutions may face escalating coordination and management costs. Although much of the existing literature has examined these relationships outside the healthcare industry, our findings echo prior health economics studies that highlight the dual nature of scale effects in healthcare provision. For instance, Söderberg and Vesterberg (58) emphasize that larger scale reduces costs, yet they overlook the possibility that excessive scale may actually increase costs. By contrast, Wang and Zhao (59) shed light on the complexities of economies of scale, supporting the interpretation of the results of this study.

Second, our results indicate that industry competition primarily moderated the right-hand side of the U-shaped relationship between gross floor area and initial operating budgets. This study provides novel evidence on how industry competition moderates the relationship, enriching the theory of industrial economics. In a highly competitive environment, long-term care institutions face stronger efficiency pressures than in low competitive environment, which limits their willingness or ability to increase investment when expanding beyond the optimal size. This reduces the incremental rise in initial operating budgets for very large facilities but does not affect the overall curvature of the U-shaped relationship, as smaller institutions still require sufficient resources to cover essential functions. In addition, when expansion reaches a certain level, competition makes additional investment difficult to translate into returns. While competition has been shown to contribute to better quality of outpatient care in hospital industry (60), this study shows industry competition negatively influenced initial operating budget of long-term care institutions. In other words, industry competition itself exerts a constraining effect on initial operating budget. The possible reason may be this: When industry competition becomes more intense, investors become more risk-aware, which result in a decline in the initial operating budget.

Third, staffing arrangement is positively associated with budget, consistent with labor economics. A higher proportion of professional and technical staff implies not only higher wage bills but also a higher baseline of human capital investment, reflecting quality-oriented production functions in services. This aligns with prior research emphasizing that labor intensity is a key driver of cost (61). Resources for health skill mix have a critical role in adherence to healthcare quality standards. Inadequate or poorly skilled staff can negatively affect delivery of quality healthcare services (62). At the same time, staffing levels influence financial sustainability in home-based long-term care facilities (63).

Fourth, ownership type exhibited significant effects. Publicly constructed institutions required lower initial operating budgets compared to privately constructed ones, likely due to subsidies, or capital support, consistent with property rights economics theories. Conversely, publicly operated institutions demanded higher budgets than private operators. Our findings suggest that institutions which are publicly constructed but privately operated appear to be the most efficient, while those which are privately constructed but publicly operated are the least efficient. Similarly, institutions with self-owned property required higher initial budgets than those with leased property. These results resonate with and extend prior literature on long-term care efficiency or cost. For example, Herr (64) finds that private and non-profit organizations are on average less cost efficient and less technically efficient than are publicly owned ones, suggesting that efficiency outcomes can vary by context. Ullmann (35) further emphasizes that organizational form, size, location, resident mix, and quality are closely related to operating costs in long-term care facilities. Taken together, our results provide additional empirical evidence for optimizing ownership arrangements in long-term care institutions.

Fifth, the service type has no significant effect on the initial operating budget. That is, the impact of providing services for persons with severe disabilities and/or dementia on initial operating budgets is not significant. A possible reason is that their additional costs are either absorbed by existing resources or offset by subsidies and policy support. Consequently, institutions offering these services do not necessarily require higher initial operating budgets compared to those without such services.

### 5.2 Limitations and directions for future research

Several limitations of this study should be acknowledged. First, despite robust empirical design, the possibility of omitted variable bias cannot be ruled out. While we considered various explanatory factors, other important variables—such as staff salary, resident mix, and management competence at the time of opening—were not included due to data limitations. Their omission may bias coefficient estimates, particularly in nonlinear specifications. To mitigate such concerns, this study conducted multiple robustness checks and endogeneity tests. The results consistently support the main findings, indicating that our conclusions are stable and reliable. Nevertheless, future studies should incorporate these and potentially other important variables. Second, gross floor area can be subdivided. Due to data constraints, we could not analyze these aspects in depth. Future research should examine whether the design of different functional areas such as care areas, public areas, administrative areas, etc. has varying impacts on each specific part of the budget. Third, we only studied the initial operating budget, focusing on the establishing phases. However, long-term care institutions also incur operational and maintenance costs, which play a crucial role in their sustainability (65, 66). Future research should explore the factors affecting ongoing operational expenses. Fourth, the classification of ownership types can be further refined. Publicly constructed care institutions can be further categorized based on the source of funding, such as funds from municipal government, district government, and sub-district government. Privately constructed care institutions can also be further subdivided based on their funding source. Likewise, publicly operated and privately operated care institutions can be further subdivided. Future research can explore how ownership type will influence the initial operational budget. Finally, the scope of application of the conclusions is limited. The sample data was limited to Shanghai, China, making it uncertain whether the findings can be generalizable to other cities or regions. Future research need to expand the scope of the sample by incorporating relevant data from other regions in order to ensure the generalizability of the findings.

### 5.3 Implications

The findings provide actionable guidance for policymakers, investors, and managers. Detailed numerical calculations are presented in the appendix.

#### 5.3.1 Implications for policymakers

First, the observed U-shaped relationship between gross floor area and initial operating budgets suggests that “one-size-fits-all” approach may be ineffective. Policymakers should prioritize medium-sized long-term institutions with gross floor area around 906–1,359 square meters, where initial operating budgets near the minimum values predicted by Model 2 occurs (see Appendix calculations 1). Furthermore, in highly competitive markets, the effect of gross floor area on initial operating budgets diminishes for larger institutions, suggesting that large-scale long-term care institutions should not be the primary focus of support, while medium- and even small-sized institutions should be prioritized. Second, ownership-related effect highlights the role of property rights in shaping initial budgetary requirements. Expanding access to public-private partnerships could reduce initial operating budget, as publicly constructed but privately operated institutions demonstrate the least budget (see Appendix calculations 2).

#### 5.3.2 Implications for investors

First, investors can use the estimated coefficients from our regression models to predict the required initial operating budgets for long-term care institutions (see Appendix calculations 3 and calculations 4). The U-shaped relationship suggests that the optimal gross floor area is 1132.5 square meters for minimizing initial operating budget requirements (see Appendix calculations 1). Minimum initial operating budgets vary by ownership type, ranging from CNY 74,960 to CNY 318,000 (see Appendix calculations 2). These insights help investors reduce uncertainty, identify efficient ownership arrangements, and avoid excessive capital commitments when entering the long-term care market. Second, when industry competition is intense, the diminishing effect of larger gross floor area on initial operating budgets suggests that investors should avoid blindly pursuing scale expansion.

#### 5.3.3 Implications for managers

Managers should monitor staffing composition and industry competitive intensity to adjust financial planning accordingly. First, managers should monitor and adjust staffing to ensure regulatory compliance. On this basis, managers can adjust the ratio of professional and technical staff to total employee. The reason is that a higher proportion of professional and technical staff increases wage bills and overall budget requirements but also enhances the institution’s human capital base. Second, in highly competitive areas, managers can allocate resources toward specialized services or diversifying services without major concerns about budget overruns. Because our finding shows offering services for severe disabilities and/or dementia does not require significantly higher initial operating budget.

### 5.4 Global relevance and contributions

Although this study is based on data from Shanghai, its findings contribute to the global literature in several ways. First, the study uncovers the relationship between structural characteristics of long-term care institutions and their initial operating budget, thereby enriching empirical evidence on the allocation of care resources. The findings are likely to apply across diverse health systems facing population aging. Second, the study integrates health, labor, property rights, and industrial economics, providing novel insights into how organizational structures shape initial operating budgets, with implications for diverse long-term care systems worldwide.

## 6 Conclusion

This study demonstrates that the initial operating budgets of long-term care institutions are significantly associated with their structural characteristics. To elaborate, gross floor area has a significant U-shaped relationship with initial operating budget. Industry competition negatively moderates this effect and also exerts a direct negative effect on initial operating budget. Staffing arrangement significantly impacts initial operating budget and ownership type plays a critical role. Publicly constructed but privately operated institutions are the most efficient, considering both construction and operation dimension. The nature of service premises significantly affects the initial operating budget, with leased properties requiring lower initial operating budgets compared to self-owned properties. In addition, long-term care institutions in urban areas are associated with slightly higher initial operating budgets than those in suburban areas. These findings highlight the importance of structural factors in initial operating budgets and provide guidance for policymakers, investors and managers aiming to optimize resource allocation in long-term care.

## Data Availability

The raw data supporting the conclusions of this article will be made available by the authors, without undue reservation.
