# Supplementary material for: The relationship between structural characteristics of long-term care institutions and their initial operating budgets
Source: Front Public Health. 2025 Oct 2;13:1669451. doi: 10.3389/fpubh.2025.1669451 (PMC12528046; doi:10.3389/fpubh.2025.1669451)
Supplement: Supplementary file 1 [file Data_Sheet_1.docx]

**Appendix detailed calculations 1:** The optimal numerical values of gross floor area

In the formula (3), if $\beta_{5}>0$, the standard method for finding the extreme value is to take the partial derivative with respect to X_1_ and set it equal to zero.

$$\frac{\partial Y}{\partial X_{1}}=\beta_{1}+2\beta_{5}X_{1}$$

$$\beta_{1}+2\beta_{5}X_{1}=0$$

$$X_{1}=-\frac{\beta_{1}}{2\beta_{5}}$$

The estimated coefficients from Model 2 (Table 6) are substituted into Equation (3) to derive the corresponding estimated equation.

$LnIOB=3.274+0.492(LnGFA-LnGFA_{mean})+2.510[Ln(SA+1)-Ln(SA+1)_{mean}]-0.474Construction+0.565Operation+0.191(LnGFA-LnGFA_{mean})^{2}$When in the following context, the extreme value emerges.

$$LnGFA-LnGFA_{mean}=-\frac{0.492}{2\times0.191}=-1.288$$

In our sample, we know $LnGFA_{mean}=8.32$

Therefore, we can continue to calculate.

$$LnGFA=-1.288+8.32=7.032$$

$$GFA=e^{7.032}=1132.5$$

In order to accommodate practical considerations, we provide policymakers with an interval range of gross floor area. If we predict gross floor area ±20%, the lower limit and upper limit are 906 and 1359 square meters, respectively.

$$1132.5\times(1-20\%)=906$$

$$1132.5\times(1+20\%)=1359$$

**Appendix detailed calculations 2:** The calculated minimum initial operating budgets under various ownership types

Suppose staffing arrangement is the minimum zero, that is, there is no professional and technical staff.

From the sample, we can know $Ln(SA+1)_{mean}=0.097$

$\begin{matrix} LnIOB=3.274+0.492\times(7.032-8.32)+2.510[Ln(0+1)-0.097]-0.474Construction+0.565Operation+0.191\times(7.032-8.32)^{2} \\ \end{matrix}$ After simplification, the formula is:

$$LnIOB=2.711-0.474Construction+0.565Operation$$

When long-term care institutions are publicly constructed but privately operated,

$Construction=1$ and $Operation=0$

$$LnIOB=2.711-0.474\times1=2.238$$

$IOB=e^{2.238}=9.37$(ten thousand yuan),that is, CNY93,700.

When long-term care institutions are publicly constructed and publicly operated,

$Construction=1$ and $Operation=1$

$$LnIOB=2.711-0.474\times1+0.565\times1=2.803$$

$IOB=e^{2.803}=16.48$(ten thousand yuan) ,that is, CNY164,800.

When long-term care institutions are privately constructed and privately operated,

$Construction=0$ and $Operation=0$

$$LnIOB=2.711$$

$IOB=e^{2.711}=15.06$(ten thousand yuan), that is, CNY150,600.

Although in practice our sample does not include cases of privately constructed but publicly operated institutions, such a type still exists in theory. When long-term care institutions are privately constructed and publicly operated,

$Construction=0$ and $Operation=1$

$$LnIOB=2.711+0.565\times1=3.276$$

$IOB=e^{3.276}=26.50$(ten thousand yuan), that is, CNY265,000.

Therefore, among all ownership types, publicly constructed but privately operated institutions were the most efficient, with the least initial operating budget CNY 93,700. Privately constructed but publicly operated institutions were the least efficient, with the least initial operating budget CNY 265,000.

In order to accommodate practical considerations, we provide investors with an interval range. If we predict initial operating budget ±20%, the lower limit and upper limit are CNY 74,960 and CNY 318,000, respectively.

$$93700\times\text{（1-20\%）=74960}$$

$$265000\times\text{(1+20\%)=318000}$$

**Appendix detailed calculations 3:** Estimating initial operating budgets with Model 2 coefficients

Suppose gross floor area is 5000 square meters, Staffing arrangement is 30%, that is, the ratio of professional and technical staff to total employee is 30%.Ownership type is privately constructed and privately operated. In our sample, we know that: $LnGFA_{mean}=8.32$, and $Ln(SA+1)_{mean}=0.097$

$LnIOB=3.274+0.492(Ln5000-8.32)+2.510[Ln(30\%+1)-0.097]+0.191(Ln5000-8.32)^{2}$ We know that:

$Ln5000=8.518$, $Ln1.3=0.262$

Therefore,

$$LnIOB=3.274+0.492\times(8.518-8.32)+2.510\times[0.262-0.097]+0.191\times(8.518-8.32)^{2}$$

$$LnIOB=3.274+0.492\times0.198+2.510\times0.165+0.191\times0.198^{2}$$

$$LnIOB=3.793$$

$IOB=e^{3.793}=44.40$(ten thousand yuan), that is, CNY444,000.

**Appendix detailed calculations 4:** Estimating initial operating budgets with Model 6 coefficients

Suppose gross floor area is 5000 square meters, Staffing arrangement is 30%, that is, the ratio of professional and technical staff to total employee is 10%.Ownership type is privately constructed and privately operated. Institution is with leased service premises. The institution is situated in suburban area. Care provided for persons without severe disabilities and/or dementia in the institution.

$LnGFA_{mean}=8.32$, and $Ln(SA+1)_{mean}=0.097$

$LnIOB=3.811+0.990\times(Ln5000-8.32)+2.534[Ln(30\%+1)-0.097]-0.496Constru+0.439Opera-0.430InduC-0.600(Ln5000-8.32)InduC+0.333(Ln5000-8.32)^{2}-0.234\times(Ln5000-8.32)^{2}-0.348\times1$ $Ln5000=8.518$, $Ln1.3=0.262$

$LnIOB=3.811+0.990\times(8.518-8.32)+2.534\times(0.262-0.097)-0.496Constru+0.439Opera-0.430InduC-0.600\times(8.518-8.32)InduC+0.333\times(8.518-8.32)^{2}-0.234\times(8.518-8.32)^{2}-0.348\times1$ Simplifying this, we can get:

$$LnIOB=3.594-0.5579InduC$$

If $InduC=1$, that is, when the institutions established, the number of long-term care institutions per 10,000 registered permanent residents aged 60 and above is 1.

$$LnIOB=3.036$$

$IOB=e^{3.036}=20.09$(ten thousand yuan), that is, CNY 200,900.

If $InduC=1.12$, when the institutions established, the number of long-term care institutions per 10,000 registered permanent residents aged 60 and above is 1.12.

$$LnIOB=3.594-0.5579\times1.12=3.594-0.625=2.969$$

$IOB=e^{2.969}=19.47$(ten thousand yuan), that is, CNY194,700.

If $InduC=0.06$, when the institutions established, the number of long-term care institutions per 10,000 registered permanent residents aged 60 and above is 0.06.

$$LnIOB=3.594-0.5579\times0.06=3.594-0.033=3.561$$

$IOB=e^{3.561}=35.05$(ten thousand yuan), that is, CNY 350,500.
